# Necrotizing fasciitis from an iliopsoas muscle abscess caused by a toothpick: A case report and literature review

**DOI:** 10.1016/j.ijscr.2020.10.009

**Published:** 2020-10-08

**Authors:** Wei-Quen Tee, Yin-Lun Chang, Pao-Jen Kuo, Chih-Hsiung Kang

**Affiliations:** a Department of Urology, Chang Gung Memorial Hospital, Kaohsiung Medical Center, Chang Gung University College of Medicine, Kaohsiung, Taiwan; b Department of Plastic Surgery, Chang Gung Memorial Hospital, Kaohsiung Medical Center, Chang Gung University College of Medicine, Kaohsiung, Taiwan

**Keywords:** Case report, Iliopsoas muscle abscess, Necrotizing fasciitis, Sepsis, Ingested toothpicks

## Abstract

**Introduction:**

Psoas muscle abscess is rare and can become more complicated to treat after they have progressed to necrotizing fasciitis. The data of secondary psoas muscle abscess cause by ingested toothpick are limited in the literature. We have done an extensive literature review and found a number of 8 cases (including our new case) of ingested toothpicks causing iliopsoas muscle abscess.

**Presentation of case:**

We present a 70-year-old man with unremarkable medical history experienced left flank pain for several days with radiated to left thigh and unable to walk. He initially exhibited drowsiness at emergency department with fever and chillness. Computed tomography showed iliopsoas abscess and necrotizing fasciitis. This patient received emergent surgical debridement and a toothpick was found lodged in the deep portion of the left psoas muscle. He was tolerated to the treatment and discharged on 58 days after the operation.

**Discussion:**

A review of the literature revealed only eight reported cases since 1946 (including ours) of ingested toothpicks migrating into the iliopsoas muscle and causing abscess formation or necrotizing fasciitis. Three of the cases did not exhibit gut perforation, possibly because of self-healing of the wound. Gastrointestinal symptoms are not always apparent when the perforation site is over the retroperitoneal space. Thorough debridement is essential if the origin of infection is unknown.

**Conclusion:**

Ingestion of a foreign body may be asymptomatic, the present case and a review of the literature indicated that ingested toothpicks can cause severe morbidity or even mortality. The diagnosis of psoas abscesses associated with toothpicks is difficult, and such cases should not be overlooked. Appropriate early surgical intervention is recommended.

## Highlights

• Ingested toothpick caused gut injury are a rare event, but caused retroperitoneal infection are relatively rare.
• The diagnosis of psoas abscesses associated with toothpicks is challenging and should not be overlooked.
• Gastrointestinal symptoms are uncommon when the gut perforation site is over the retroperitoneal space.
• Appropriate early surgical intervention is recommended. Thorough debridement is essential if the origin of infection is unknown.

## 1 Introduction

The iliopsoas muscle abscess is rare and categorized as primary or secondary abscesses. The infection source of primary abscesses is often unknown but these abscesses are believed to be generally caused by a hematogenous spread of infection. Secondary psoas abscesses denote cases in which the causative event or disease is known and the infection has spread from another anatomical structure [[1], [2], [3]]. The classic triad of signs and symptoms of psoas abscesses is fever, flank pain and limited hip movement (typical psoas sign). The mortality rate of psoas abscesses can reach 100% if left undiagnosed and untreated [1]. Moreover, psoas abscesses become more complicated to treat after they have progressed to necrotizing fasciitis [4]. The data of secondary psoas muscle abscess cause by ingested toothpick are limited in the literature. We have done an extensive literature review and found a number of 8 cases (including our new case) of ingested toothpicks causing iliopsoas muscle abscess. This project has been reported in line with the SCARE criteria [5].

## 2 Case presentation

A 70-year-old man presented to the emergency department after experiencing left lower back pain for several days. He had a history of benign prostate hyperplasia, perforated peptic ulcer status post operation 10 years ago. He denied any remarkable family history, medication history, and psychosocial history.

**Fig. 1:**
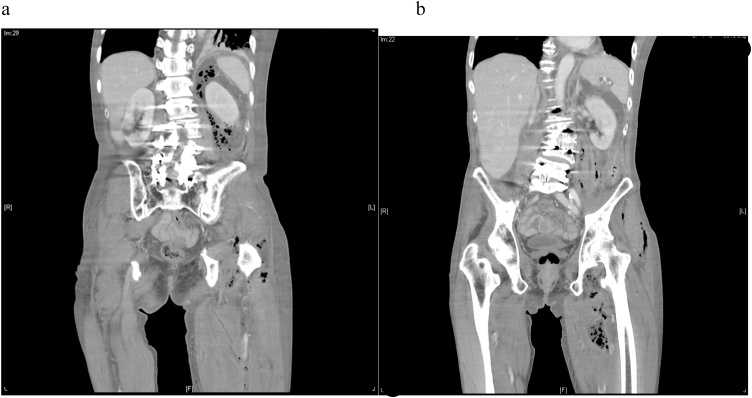
a, b: Fluid accumulation with air formation over the left iliopsoas muscle that extended into the retroperitoneum, left pelvic cavity and left thigh region.

The patient had experienced left flank pain for several days. The pain radiated to the left thigh and he reported being unable to walk for approximately 2 to 3 days. In addition, he experienced intermittent fever and chills. He denied any recent trauma, painful micturition, or hematuria. He initially went to a local hospital for medical treatment, where he received antibiotics. However, his symptoms progressed. He was then referred to our emergency department. His vital signs on admission were as below: Temperature = 37.2 °C, blood pressure = 122/71 mmHg, heart rate = 98 beats per minute, respiratory rate = 19 cycles per minute. He initially exhibited drowsiness. Physical examination revealed tenderness in the left lower abdomen and swelling in the left thigh, with limited range of motion. No external wounds, erythema, localized heat, or blister lesions were noted. Laboratory data indicated leukocytosis (13.1k/uL) with a left shift (neutrophils 89%) and bandemia (8%), severe coagulopathy (international normalized ratio >5), elevated C-reactive protein (325 mg/L), hyperlactatemia (46 mg/dL), and acute kidney injury (with an increase in creatinine from 0.94 mg/dL to 1.34 mg/dL). Computed tomography (CT) of the abdomen and lower limbs revealed fluid accumulation over the left iliopsoas muscle that extended into the retroperitoneum, left pelvic cavity, and left thigh region, which suggested an iliopsoas abscess and necrotizing fasciitis (Fig. 1a and b). The initial impression was sepsis with disseminated intravascular coagulation. Upon observation of the left iliopsoas abscess and necrotizing fasciitis extending to the left thigh, we performed blood culture and initiated empirical antibiotic therapy with 1 g of ertapenem once a day and 1 g of vancomycin every 12 h.

## 3 Treatment and outcome

**Fig. 2:**
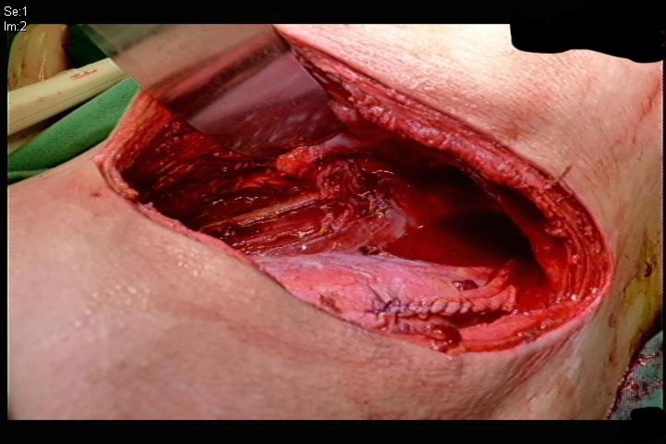
Left flank incision wound after debridement and pus drainage.

**Fig. 3:**
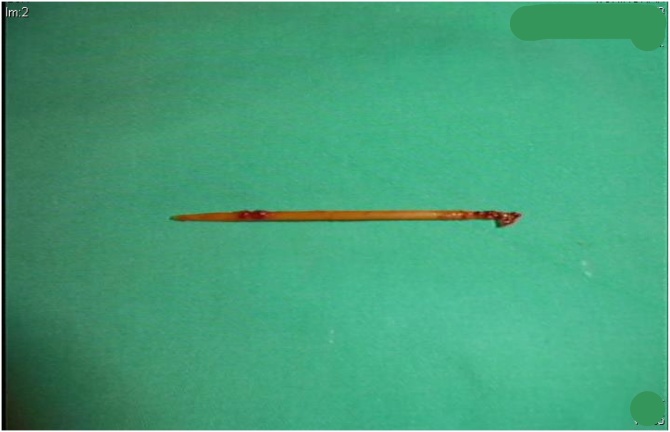
A toothpick was found over the left deep segment of the psoas muscle during surgical debridement.

After correcting the coagulation, urology senior resident combined with plastic surgeon performed emergency debridement and fasciotomy. A left flank retroperitoneal incision was made. Copious amounts of malodorous pus and some slough and necrotic tissues were drained from the psoas muscle (Fig. 2). We found a foreign body, identified as a toothpick, lodged in the deep portion of the left psoas muscle (Fig. 3). No ascites or bowel contents were observed in the retroperitoneal space. The patient did not experience gastrointestinal symptoms prior to the operation. After the operation, he was admitted to the surgical intensive care unit. The pus culture showed aerobic (Streptococcus anginosus) and anaerobic (Fusobacterium varium, Solobacterium moorei) bacteria. We continued antibiotic treatment according to the antibiotic susceptibility of the bacteria. During his stay in the surgical intensive care unit, the patient received regional fasciotomy and debridement of the left thigh region another three times. He was transferred to an ordinary ward 25 days after the operation and discharged 58 days after the operation. He was tolerated to the treatment and able to walk as previous.

**Fig. 4:**
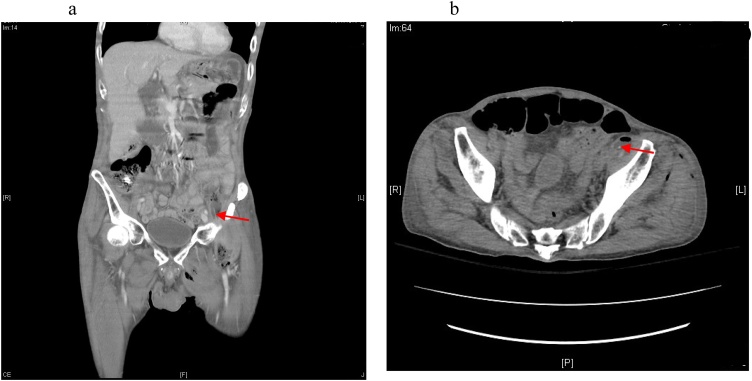
a, b: The red color arrow points to the toothpick. The toothpick was approximately 40–60 Hounsfield units.

According to the patient’s family, he had a habit of chewing on toothpicks. We suspected that he had swallowed a toothpick by accident and that extraluminal migration of the toothpick caused abscess formation. The toothpick was identified upon review of the abdominal CT (Fig. 4a and b) over the left retroperitoneal space. The series of CT images showed that the toothpick was approximately 40–60 Hounsfield units.

## 4 Discussion and literature review

The effects of swallowing a foreign body can vary from asymptomatic to life-threatening. Inflammation, reactive fibrosis, and perforation can occur if the foreign body fails to pass through the digestive system. Steinbach et al. [6] reported that approximately 80% of patients who accidentally ingested toothpicks experience perforation of the gastrointestinal tract. The toothpick may also migrate to and become lodged in adjacent structures, most commonly the liver, followed by the retroperitoneal space. Because of its presentation as atypical and nonspecific symptoms, psoas infection in the retroperitoneal space can mimic the presentation of other diseases, leading to a delayed diagnosis. Most importantly, toothpicks are often radiolucent, making them difficult to detect through CT; thus, diagnosis of psoas abscesses caused by toothpicks is challenging.

A review of the literature revealed only eight reported cases since 1946 (including ours) of ingested toothpicks migrating into the iliopsoas muscle and causing abscess formation or necrotizing fasciitis (Table 1). All of the cases were men aged over 40 years. Including ours, three of the cases did not exhibit gut perforation, possibly because of self-healing of the wound. Most of the cases did not involve gastrointestinal symptoms such as abdominal pain. Gastrointestinal symptoms are not always apparent when the perforation site is over the retroperitoneal space. In one of the cases, the psoas abscess recurred after multiple episodes of pigtail drainage [7]. The patient recovered after removal of the toothpick through debridement. A advantages of percutaneous drainage are that general anesthesia and the stress of surgery can be avoided and it is suitable for well-defined unilocular abscesses [8]. However, surgical drainage is recommended if percutaneous drainage fails or in cases of multilocular abscesses or considerable involvement of adjacent structures [3]. In almost all of the reported cases, the toothpick was lodged in the peri abscess region. However, if the area of necrotic tissue is broad, the foreign body is difficult to locate. In our case, we performed debridement until no ongoing necrosis was evident and unintentionally found the toothpick. Thorough debridement is essential if the origin of infection is unknown. Two of the patients reported in the literature died due to severe sepsis, and all of them underwent surgical debridement and antibiotic treatment.

**Table 1:** Summary of eight reported cases of peri-psoas abscesses caused by ingested toothpicks.

Year	Author	Age	Sex	Hospital course	Source	Site of toothpick abscess	Bacteriology	GI s/s	Event recall
1946	M.B Landers et al. [11]	56	M	Fever with right lumbar pain 5 days after herniorrhaphy. Incision and drainage were performed. The patient recovered within 2 months	Posterior wall of the ascending colon (suspected)	Right perinephric space	Gram negative rods and short chained streptococci	No	No recollection
1969	Robert D. Shaffer et al. [12]	51	M	Admitted semicomatose with subcutaneous emphysema in the right thigh, Incision and drainage were performed and traced to the retroperitoneal space. The patient died 52 h after admission.	Malrotation of the colon with terminal ileum 20-cm site perforation (autopsy)	Fistulous tract from the ileum to the right iliopsoas muscle	Escherichia coli, Aerobacter aerogenes, haemolytic streptococci, and Clostridium perfringens	Diarrhea and vomiting 5 days prior to admission that later subsided	Habit of chewing of toothpick at work
1992	Brett D. Archer et al. [13]	59	M	Right iliac fossa pain and fever. Pain radiated to the thigh and patella. Laparotomy and drainage were performed. The patient was discharged 9 days post operation.	second part of duodenal perforation	Right psoas muscle	Streptococcus milleri and Streptococcus morbillorum	No	Recalled eating a filet mignon containing wooden skewers 2 weeks previously, wore dentures
2000	Johannes Zacherl et al. [7]	69	M	Right abdominal pain for 3 months, CT showed right psoas abscess, which recurred after drainage. Surgical debridement was performed.	Scar tissue between the abscess wall and inferior duodenum	Right psoas muscle	Escherichia coli and enterococci	Right abdominal pain	n/a
2003	N. Lellouche et al. [14]	67	M	Fever, painful swollen left thigh with complete disability. Surgical exploration, debridement, and colostomy were performed. The patient died 10 days post operation.	Rectosigmoid colon perforation	Left pericolic abscess to left thigh	Escherichia coli, Streptococcus constellatus, and Bacteroides thetaiotaomicron	No	No history of toothpick ingestion
2011	I-Hsin Lee et al. [15]	41	M	Right hip pain for 2 weeks. The right hip exhibited local erythema with crepitation and right lower quadrant abdominal tenderness. Debridement was performed. The patient recovered.	Terminal ileum perforation	Right pelvic region along iliopsoas muscle into buttock	Bacteroides fragilis, Escherichia coli, and Prevotella spp.	No	No recollection
2018	Markus Rupp et al. [4]	51	M	Gas gangrene in the right lower abdomen and right leg. Surgical debridement was performed. The patient recovered within 2months.	Sigmoid colon perforation	Retroperitoneum and right thigh with gluteus muscle and hip abductors	Extended-spectrum beta-lactamase producing Escherichia coli	n/a	No recollection
2018	Our case	70	M	Left lower back pain radiating to the left thigh, inability to walk for days, impending septic shock. CT showed necrotizing fasciitis. Thorough surgical debridement was performed. The patient was discharged 2 months post operation.	Gastrointestinal tract (suspected)	Deep segment of the left psoas muscle	Streptococcus anginosus, Fusobacterium varium, Solobacterium moorei	Left lower abdominal tenderness	Habit of chewing toothpicks

The abscess sites of seven of the cases, including ours, were over the right side. In addition, the sites of gut perforation were mostly on the right side, including the duodenum, terminal ileum (ileocecal region), and ascending colon. Retroperitoneal abscesses can spread to the lower extremities through two routes: through the sciatic foramen to the buttock and hip or through the obturator or femoral canal to the thigh and hip [3,9]. Few of the reported patients recalled swallowing the toothpick, and a migrating toothpick may be asymptomatic until infection occurs. Toothpicks or skewers may be accidentally ingested with food at mealtimes, especially when consuming alcohol or wearing dentures, both of which dull sensation in the palate [10].

In conclusion, this paper reports a very rare case of an ingested toothpick causing a psoas abscess with progression to necrotizing fasciitis. Although ingestion of a foreign body may be asymptomatic, the present case and a review of the literature indicated that ingested toothpicks can cause severe morbidity or even mortality. The diagnosis of psoas abscesses associated with toothpicks is difficult, and such cases should not be overlooked. Appropriate early surgical intervention is recommended. Until the foreign body is removed, exploration of the origin of the abscess and debridement are crucial. Therefore, clinicians should keep in mind that this rare condition is also a challenge for surgeons.

## Declaration of Competing Interest

All authors have nothing to disclose.

## Funding

All authors have no source of funding to disclose.

## Ethical approval

There is no ethical approval was obtained as it’s a case report.

## Consent

Written informed consent was obtained from the patient for publication of this case report and accompanying images. A copy of the written consent is available for review by the Editor-in-Chief of this journal on request.

## Author contribution

Study concepts: Wei-Quen Tee, Yin-Lun Chang.

Study design: Wei-Quen Tee, Yin-Lun Chang.

Data acquisition: Wei-Quen Tee.

Quality control of data and algorithms: Yin-Lun Chang, Chih-Hsiung Kang.

Data analysis and interpretation: Wei-Quen Tee, Yin-Lun Chang.

Manuscript preparation: Wei-Quen Tee.

Manuscript editing: Wei-Quen Tee, Yin-Lun Chang.

Manuscript review: Chih-Hsiung Kang, Pao-Jen Kuo.

## Registration of research studies

Our paper is a case report, no registration was done for it.

## Guarantor

Chih-Hsiung Kang, chkang5801@gmail.com

## Provenance and peer review

Not commissioned, externally peer-reviewed.
